# A Report of Candida blankii Fungemia and Possible Endocarditis in an Immunocompetent Individual and the Review of Literature

**DOI:** 10.7759/cureus.14945

**Published:** 2021-05-10

**Authors:** Vidya S Kollu, Pramod K Kalagara, Shehla Islam, Asmita Gupte

**Affiliations:** 1 Department of Infectious Diseases and Global Medicine, College of Medicine, University of Florida, Gainesville, USA; 2 Department of Hospital Medicine, Covenant Healthcare, Saginaw, USA; 3 Division of Infectious Diseases, Veterans Affairs Medical Center, Gainesville, USA

**Keywords:** candida blankii, fungal endocarditis, fungemia, antifungal resistance, antifungal duration

## Abstract

*Candida blankii* is an emerging pathogenic fungus, first identified in 1968 as a new species. In the past five years, it has been identified in cystic fibrosis patient's airways and as fungemia in immunocompromised patients (post lung transplant and preterm neonates). It has been postulated to be a possible opportunistic pathogen based on the published case reports. We report a case of *C. blankii* fungemia with possible endocarditis in an immunocompetent individual. To our knowledge, this is also the first case of *C. blankii* bloodstream infection reported in an adult patient (age > 18 years). The *C. blankii *isolate from our patient had high minimum inhibitory concentrations (MICs) to azoles similar to the published reports. There is a dearth of literature guiding the treatment of this organism, given the variable susceptibility pattern and lack of data. Here, we describe successful treatment of possible *C. blankii* endocarditis with a combination of polyene and echinocandin antifungal agents.

## Introduction

*C. blankii* is an emerging fungal pathogen [1]. It has been reported as a cause of infection of the airways in cystic fibrosis patients and as a cause of fungemia in immunocompromised patients in the last five years [2-5]. We report a case of *C. blankii* fungemia with possible endocarditis in an immunocompetent individual. This case would also be the first case of *C. blankii* infection reported in an adult patient (age > 18 years). Antifungals should be chosen based on the sensitivities as the organism is known to be resistant to some antifungals.

## Case presentation

**Figure 1:**
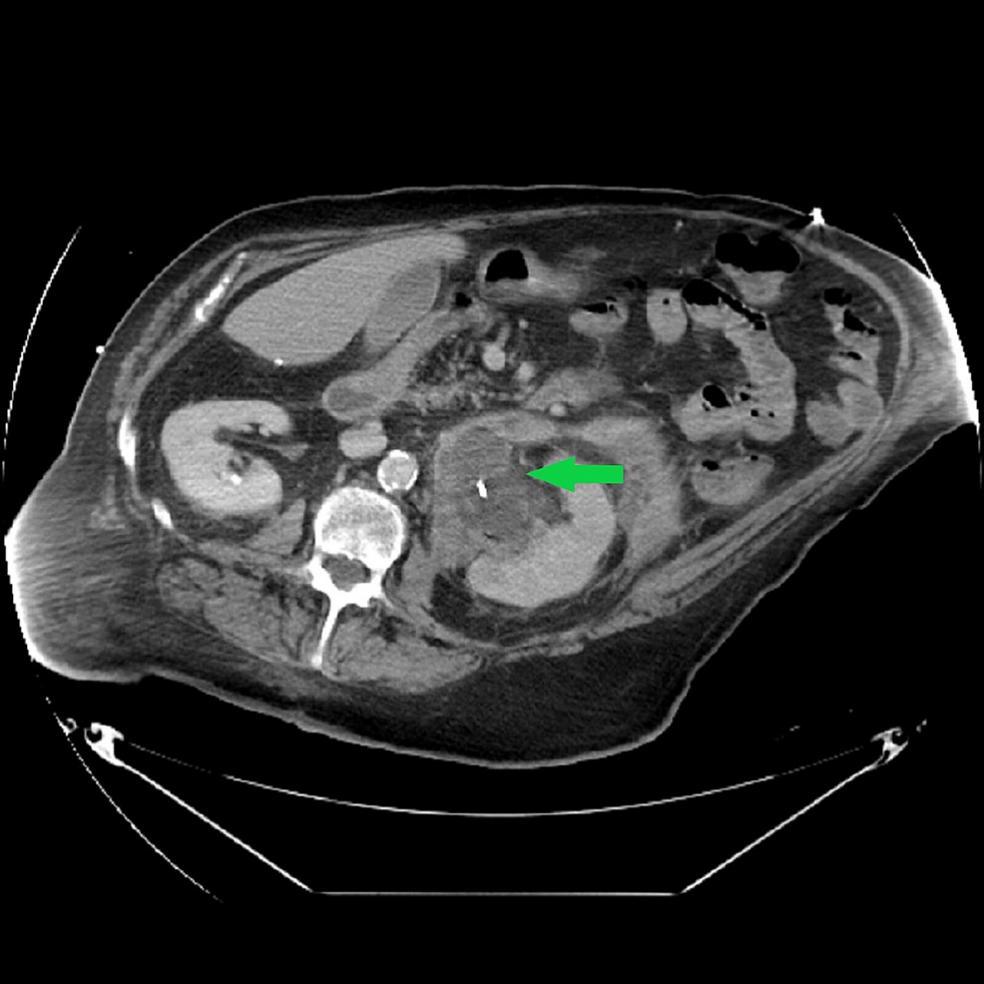
CT Abdomen and Pelvis Showing Renal Calculi at the Distal Left Ureter Causing Severe Left-sided Hydroureteronephrosis. There is a Multiloculated Fluid Collection in the Left Perinephric Space Representing Possible Perinephric Abscess.

A 63-year-old male with a history of hypertension, hyperlipidemia, diabetes mellitus, gastroesophageal reflux disease, cerebrovascular accident, and chronic low back pain was admitted with sepsis. He was diagnosed with obstructing left ureteral calculi and perinephric abscess (Figure 1).

**Figure 2:**
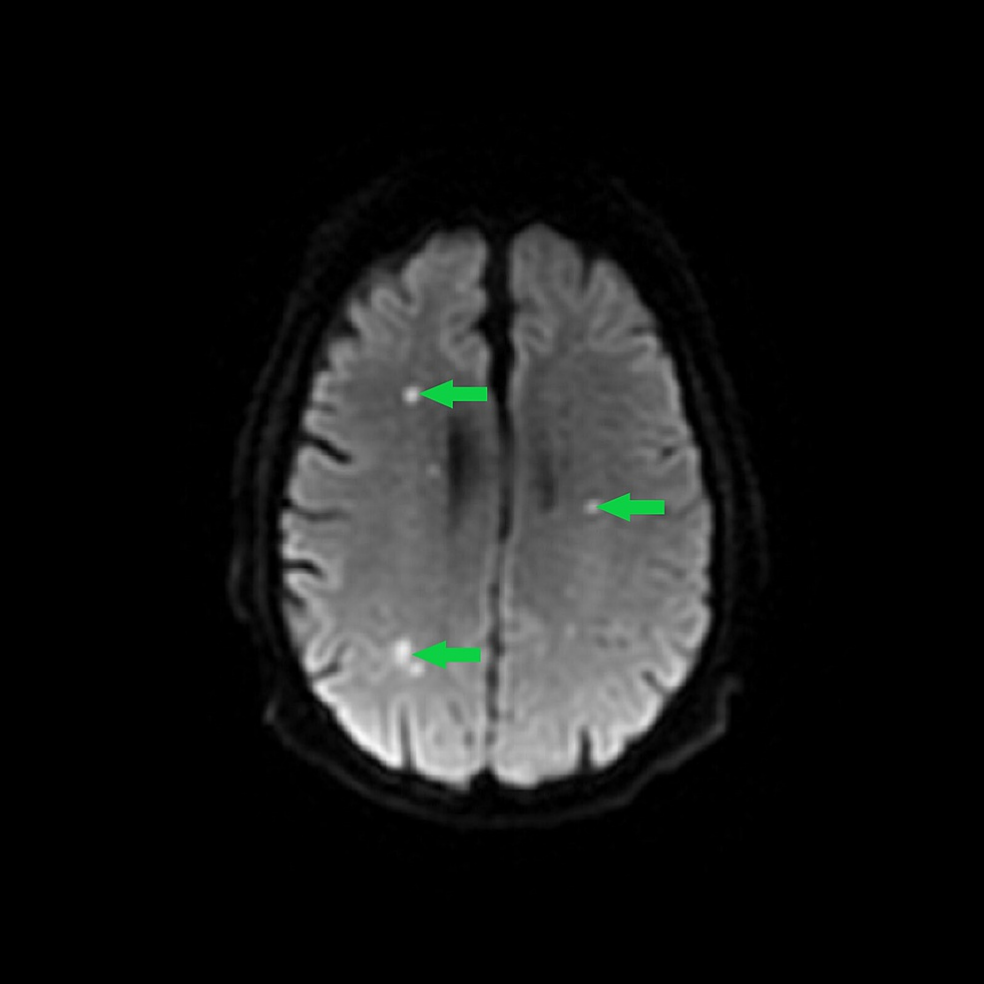
MRI Brain Showing Multiple Scattered Bilateral Foci of Restricted Diffusion Consistent With Acute Embolic Infarctions.

He underwent ureteral stent placement and drainage of the abscess. Abscess cultures grew *Candida albicans*. The patient was started on broad-spectrum antibiotics (vancomycin and aztreonam) on admission to treat sepsis, and he received them for six days before ureteral stent placement. His blood cultures remained negative, but urine culture yielded yeast which our laboratory did not identify. After clinical improvement, he was discharged on linezolid, fluconazole, and aztreonam for two weeks. Three weeks after completing the antimicrobial treatment, he was readmitted with complaints of fevers, chills and blurry vision. Physical examination was positive for left costovertebral angle tenderness and nystagmus. He was found to have elevated heart rate (99 beats/minute) and white count (18.84 K/cmm). Contrast-enhanced magnetic resonance imaging (MRI) of the brain showed multiple, scattered bilateral acute embolic shower/infarctions (Figure 2).

Blood cultures grew *Staphylococcus lugdunensis* and yeast. Our laboratory could not identify the yeast, and the isolate was sent to a reference laboratory for further identification. Urine cultures grew *Citrobacter freundii* and *methicillin-resistant Staphylococcus aureus* (MRSA). MRSA was not isolated from blood cultures. Risk factors for MRSA urinary tract infection (UTI) in this patient could have been the presence of a ureteral stent (placed during prior hospitalization) or transient bacteremia seeding the urinary tract. Transesophageal echocardiogram (TEE) showed thick interatrial septum and small mobile vegetation (1.1 cm) on the posterior mitral leaflet. There was no evidence of mitral regurgitation. Due to the presence of fungemia, ophthalmology evaluated the patient, and endophthalmitis was ruled out. Repeat blood cultures drawn at 48-hours after initiation of antimicrobials showed no growth. The cardiothoracic surgical team deemed the patient to not be an appropriate candidate for valve replacement surgery given his comorbid conditions, rapid clearance of blood cultures, and absence of signs for heart failure or mitral regurgitation.

The ureteral stent was removed. The patient was treated medically for polymicrobial endocarditis with antimicrobial coverage for *Staphylococcus lugdunensis*, MRSA (due to the possibility of transient bacteremia from urinary source), and yeast. Since the yeast species could not be identified, we initiated micafungin and liposomal amphotericin B (AmBisome). For *Staphylococcus lugdunensis* and MRSA, initially, the patient was started on intravenous vancomycin and later switched to daptomycin due to the concern for nephrotoxicity. The patient was treated with meropenem to cover *Citrobacter freundii* from the renal abscess. The patient showed clinical improvement on the above regimen. Four weeks into the treatment, the reference lab identified the yeast noted in the blood culture as *Candida blankii*, and the minimum inhibitory concentrations (MICs) for antifungals were as reported in Table 1.

**Table 1:** Sensitivities of C. blankii Isolate MIC: Minimum inhibitory concentration

Anti-Fungal	MIC
Amphotericin B	0.500 mcg/mL
Anidulafungin	0.250 mcg/mL
Caspofungin	1 mcg/mL
Fluconazole	16 mcg/mL
5-Flucytosine	<=0.060 mcg/mL
Itraconazole	0.500 mcg/mL
Micafungin	0.120 mcg/mL
Posaconazole	1 mcg/mL
Voriconazole	0.250 mcg/mL

The patient received six weeks of daptomycin and AmBisome from the day of the first negative blood cultures. He also received meropenem for four weeks duration. Micafungin which was started along with AmBisome was extended for 12 weeks, followed by which he was started on oral voriconazole for chronic suppression, which the patient continued for nine months. Nine months after his initial diagnosis of endocarditis, he is doing well with no recurrence of bacteremia or fungemia.

## Discussion

*C. blankii* as a species was first identified and described by Buckley and van Uden in 1968. It is a non-fermenting yeast identified in the organs of a mink. It was named in honor of Dr. Blank, who first identified this microorganism [1]. It has been investigated in the production of biomass and also for estimating biological oxygen demand for water/water sources [6-9]. Although *Candida* species are known to be a normal commensal on the skin and mucosa of the human body, *C. blankii* is not known to be one.

In 2015, Zaragoza et al. were the first to describe *C. blankii* as a pathogen in a 14-year-old cystic fibrosis patient with recurrent exacerbations. It was repeatedly isolated from the patient's airways. The patient showed clinical improvement with stabilization of FEV1, improvement in exacerbations, and weight gain after he was treated with itraconazole [5]. De Almeida et al. reported *C. blankii* fungemia in a 16-year-old female cystic fibrosis patient during the immediate postoperative period after a bilateral lung transplant [4]. Susceptibility testing showed high MICs to echinocandins but sensitive to Amphotericin B. They postulated that it was likely present in the airways and caused fungemia due to lack of antifungal prophylaxis. The patient was initially treated with liposomal Amphotericin B but was discontinued due to adverse effects. She was treated with micafungin for two weeks and improved clinically [4]. In 2018, Al-Haqqan et al. reported fungemia due to *C. blankii* in a 27-week preterm neonate [2]. Chowdhary et al. reported a case series of *C. blankii* fungemia in nine neonates over a seven-month period in a neonatal intensive care unit in India [3].

Our patient is the first known adult patient to be reported with *C. blankii* fungemia and possible endocarditis. Our patient was complicated as his original source of infection was urinary but subsequently developed *Staphylococcus lugdunensis* bacteremia and *C. blankii* fungemia. TEE showed findings consistent with endocarditis. We cannot say with certainty that *C. blankii* caused endocarditis as *Staphylococcus lugdunensis* can cause endocarditis as well. For *C. blankii* endocarditis diagnosis, one major (vegetation on TEE) and two minor (fever and positive blood culture that does not meet major criterion) Modified Duke's criteria were met which qualifies as possible endocarditis. Two major (vegetation on TEE and typical bacteria in two separate blood cultures) and one minor (fever) criteria were met for *Staphylococcus lugdunensis* endocarditis, thus meeting the definition of definite infective endocarditis [10]. The endocarditis could have been caused by one or other of these organisms. Although less likely, the possibility of both being etiologic organisms cannot be ruled out. Therefore, the clinical decision was made to treat the patient for endocarditis caused by both organisms. Of note, our patient had no prior significant immunosuppressive condition and his glycosylated hemoglobin was 5.8%. *C. blankii* tends to be resistant to many commonly used antifungals. Due to the paucity of infections due to this species, there are no clear recommendations on the drug of choice for *C. blankii* fungemia. Table 2 summarizes the MICs for various antifungal agents and the treatment outcomes in the published case reports/series.

**Table 2:** Summary of the Sensitivities, Treatments, and Outcomes of the Published Cases/Case Series AmpB: Amphotericin B, Ani: Anidulafungin, Cas: Caspofungin, Flu: Fluconazole, 5FC: 5 Flucytosine, Itra: Itraconazole, Mica: Micafungin, Posa: Posaconazole, Vori: Voriconazole, n/a: not available, CF: Cystic fibrosis

Case Report	Antifungal MIC (mcg/mL)									Antifungal & Duration of Therapy	Outcome
	AmpB	Ani	Cas	Flu	5FC	Itra	Mica	Posa	Vori		
Our patient	0.5	0.25	1	16	<=0.06	0.5	0.12	1	0.25	AmpB and Mica for 6 weeks, then Mica alone till 12 weeks, then Vori for 6 months	Recovered
Zaragoza et al. [5]	<=0.13	<=0.13	<=0.13	<=0.13	n/a	<=0.13	n/a	<=0.13	<=0.13	Itra 200 mg daily for 2 weeks and then 100 mg daily for suppression	Weight gain and no exacerbations of CF
De Almeida Jr. et al. [4]	0.5	1	n/a	16	n/a	n/a	0.5	n/a	0.5	Mica for 2 weeks	Survived and discharged
Al-Haqqan et al. [2]	0.125	0.19	0.25-0.5	12-16	n/a	0.75	0.125	0.5-0.75	0.19-0.38	AmpB and Flu for 2 weeks, followed by AmpB alone and then a combination of AmpB and Cas	Death from polymicrobial sepsis
Chowdhary et al. [3]	0.25-0.5	2	1-2	8	0.125	0.125-0.25	0.06-0.125	0.06-0.25	0.25	Flu for varying duration	4 deaths and 5 survived

Recommended treatment for *Candida* native valve endocarditis is amphotericin B with or without flucytosine or echinocandins, followed by azole step-down therapy. Along with this, valve replacement is strongly recommended, and antifungals should be continued for at least six weeks from the day of valve replacement or even longer if there are any associated complications. In patients who cannot undergo valve replacement, long-term suppression is advised [10]. Our patient had a successful outcome with nine months of antifungal therapy without surgery.

## Conclusions

To the best of our knowledge, we report the first case of *C. blankii* fungemia and possible endocarditis in an adult patient (18 years or older). Our patient is immunocompetent, and previously, *C. blankii* was reported as a pathogen in immunocompromised individuals (cystic fibrosis, lung transplant, and preterm neonates) only. In prior reports, sensitivities showed higher MICs for azoles, and some isolates have higher MICs for echinocandins. There was a varying success in treatment. The isolate from our patient showed similar high MICs for both azoles and echinocandins. Our patient responded well to the combination of liposomal amphotericin B and micafungin, followed by suppressive therapy with voriconazole. More studies and data are needed to understand the pathogenesis for *C. blankii* in humans, to recommend the drug of choice for treatment, and guidance for the duration of treatment for various indications. We think this case is clinically important as there is a paucity of case reports describing the clinical manifestations, treatment choices, and subsequent outcomes for *C. blankii* infections.
